# Anatomy and Surgery of the Endoscopic Endonasal Approach to the Skull Base

**Published:** 2012-01-18

**Authors:** Domenico Solari, Alessandro Villa, Michelangelo De Angelis, Felice Esposito, Luigi Maria Cavallo, Paolo Cappabianca

**Affiliations:** Department of Neurological Sciences, Division of Neurosurgery, Università degli Studi di Napoli Federico II, Naples, Italy

**Keywords:** endoscopy, skull base surgery, endoscopic endonasal surgery, endoscopic anatomy

## Abstract

The midline skull base is an anatomical area, which extends from the anterior limit of the anterior cranial fossa down to the anterior border of the foramen magnum. For many lesions of this area, a variety of skull base approaches including anterior, antero-lateral, and postero-lateral routes, have been proposed over the last decades, either alone or in combination, often requiring extensive neurovascular manipulation.

Recently the endoscopic endonasal approach to the skull base has been introduced to access the midline skull base.

The major potential advantage of the endoscopic endonasal technique is to provide a direct anatomical route to the lesion since it does not traverse any major neurovascular structures, thereby obviating brain retraction.

The potential disadvantages include the relatively restricted exposure and the higher risk of CSF leak.

In the present study we report the endoscopic endonasal anatomy of different areas of the midline skull base from the olfactory groove to the cranio-vertebral junction and accordingly describe the main features of the surgical approaches to each of these regions.

The skull base is one of the most fascinating and complex areas, either from an anatomical and a surgical standpoint; as a matter of facts it can be involved in a variety of lesions, neoplastic and/or inflammatory and/or infectious, whose successful treatment may be troublesome, being overburdened by an high grade of invasiveness, morbidity and mortality. A variety of innovative skull base cranio-facial approaches including anterior, antero-lateral and postero-lateral routes, have been developed (1–4). Recently, technical advances and scientific progress has lead to a progressive reduction of the invasiveness of these approaches and the possibility to access the skull base from the nose that gradually took place. The transsphenoidal approach developed perfectly fitting this conceptual way of thinking, being founded on the possibility of gaining access through natural preconstituted, i.e. sphenoid sinus cavity. It has been reserved for the surgical management of lesions involving the sellar and the closest areas up to 1987, when Weiss (5) termed and described the **extended transsphenoidal approach**, intending a transsphenoidal approach with removal of additional bone along the tuberculum sellae and the posterior planum sphenoidale to expose and access the suprasellar space. Later, it has been the fundamental contribute brought by the endoscope that boosted the extension of the transsphenoidal approach to the several areas of the skull base. (6–19). Though, the endoscopic endonasal surgery has been developing through innovations and technological progress to grant the lowest rates of morbidity and mortality in a safe, feasible, limited, yet practical way. It requires specific endoscopic skills, and is based on a different concept: the endoscopic view that the surgeon receives on the video monitor is not the transposition of the real image, rather consisting of a microprocessor’s elaboration process. Such a brilliant intraoperative imaging modality bringing the surgeon’s eyes close to the relevant target, has provoked favour and disagreement, for the aspects related to the different working conditions. As a matter of facts, the endoscopic technique requires different instrumentation, consisting in different components: the endoscope, the fiberoptic cable, the light source, the camera, the monitor, and the video recording system, related one to another as a “clock mechanism”. Besides, further advantages have been identified: the endoscopic endonasal technique turned out to be a lesser traumatic procedure for the nasal structures, with a minimal postoperative pain and a better relief after surgery, in the majority of cases, does not requiring any nasal packing.

The introduction of modern endoscopic techniques afforded the reinassance of the transsphenoidal route for the treatment of several different lesions of the skull base: the endoscopic technique has been though adopted according to classical indications for midline lesions and, more recently, along with surgeons’ confidence, also extending them to lesions of paramedian and lateral compartments of the ventral skull base.

## Endoscopic anatomy of the skull base: the endonasal perspective

The anatomy of the midline skull base can be divided in three areas:

- 1) the midline anterior skull base: from the frontal sinus to the posterior ethmoidal artery;
- 2) the middle skull base: the sphenoid sinus cavity;
- 3) the posterior skull base: from the dorsum sellae to the cranio-vertebral junction;

## The Anterior skull base

The endonasal view of the midline anterior skull base corresponds to the roof of the nasal cavities. After the removal of the anterior and posterior ethmoid cells and of the posterior part of the septum (lamina perpendicularis), the anterior skull base appears as a rectangular area limited laterally by the medial surfaces of the orbital walls (lamina papyracea), posteriorly by the planum sphenoidale and anteriorly by the two frontal recesses. Each part is formed by the lamina cribrosa medially and ethmoid labyrinth laterally (20). The arterial supply of the dura mater of the ethmoidal planum is ensured by the anterior ethmoidal artery (AEA) and the posterior ethmoidal artery (PEA) that are branches of the ophthalmic artery. These vessels send many small branches to the cribriform plate where they anastomize with the nasal branches of the sphenopalatine artery.

## The middle skull base

The middle skull base corresponds to the posterior and lateral walls of the sphenoid sinus, where a series of protuberances and depressions are recognizable. The sellar floor is at the center, the spheno-ethmoid planum above it and the clival indentation below; lateral to the sellar floor the bony prominences of the intracavernous carotid artery (ICA) and the optic nerve can be seen and, between them, the lateral opto-carotid recess, moulded by the pneumatization of the optic strut of the anterior clinoid process (21). Although rarely visible in the cavity of the sphenoid sinus, it is important to define the position of the medial opto-carotid recess – present in about 50% of cases – since its removal on both sides significantly increases the surgical exposure over the suprasellar area. The medial opto-carotid recess can be identified using as landmarks of access to the suprasellar space. To explore the suprasellar region, the surgeon has to remove the bone and the dura over the sella, the tuberculum sellae and the posterior part of the planum sphenoidale.

Once the dura has been opened it is possible to visualize in the **supra-chiasmatic area:** the anterior margin of the chiasm and the medial portion of the optic nerves in the chiasmatic cistern, as well as the A_1_ and A_2_ segments and the anterior communicating artery in the lamina terminalis cistern. While in the **sub-chiasmatic area** the first structure encountered is the pituitary stalk. The superior hypophyseal arteries and the branches for the inferior surface of the optic chiasm and nerves are visible.

## The posterior skull base

The midline posterior cranial fossa is represented by the anterior surface of the clivus, from the dorsum sellae to the cranio-vertebral junction. The clivus is divided by the inferior wall of the sphenoid sinus in an upper part (sphenoid portion) and in a lower part (rhino-pharyngeal portion). Laterally on the sphenoid portion of the clivus the carotid protuberances are visible. After removal of the bone of the upper part of the clivus the periostium-dural layer is exposed; the opening of the carotid protuberance permits to identify the sixth cranial nerve, which passes together with the dorsal meningeal artery just medially to the paraclival carotid artery, thus representing the real lateral limit of the approach at this level. After the opening of the dura, the basilar artery and its branches, as well as the upper cranial nerves, are well seen along their courses in the posterior cranial fossa.

The rhinopharynx is exposed extending the bone removal to the inferior wall of the sphenoid sinus. The lower third of the clivus is removed and both the *foramina lacera* are identified, representing them the lateral limit of the approach at this level. It is possible to further enlarge this opening by removing the anterior third of the occipital condyles, without entering in the hypoglossal canal, which is located at the junction of the anterior and middle third of each occipital condyle. Moreover the articular surface of the condyle is located on its lateral portion, so that the removal of its inner third, through an anterior route, does not involve the articular junction. The mucosa of the rhinopharynx is removed and the atlanto-occipital membrane, the longus capitis and colli muscles, the atlas and axis are exposed. The anterior arch of the atlas is removed and the dens is exposed. Using the microdrill the dens is thinned; it is then separated from the apical and alar ligamens and finally dissected from the transverse ligament and removed.

## Endoscopic endonasal approaches

### Basic concepts

The endoscopic endonasal procedure is performed using a rigid 0-degree endoscope, 18 cm in length 4 mm in diameter (Karl Storz Endoscopy, Tuttlingen, Germany), as the sole visualizing instrument of the surgical field; sometimes angled scope are used to further explore the suprasellar area after the lesion removal. Dedicated surgical instruments with different angled tips are needed in order to permits movements in all the visible corners of the surgical field (22, 23). A detailed, complete preoperative planning, even integrated by tridimensional computerized reconstruction of MRI and/or CT scans, has to be the backbone of the surgical procedure.

An image-guided system (neuronavigator) is also required – specially when the classic landmarks are not easily identifiable -; it provides information for midline and trajectory and offers more precision in defining the bony boundaries and the neurovascular spatial relationships. (24, 25)

Finally, it is extremely important to use dedicated instruments: different **endonasal bipolar forceps** have been designed, in order to be easily introduced and maneuvered in the nasal cavity, with various diameters and lengths. **High-speed low-profile drills**, long enough but not too bulky, may be very helpful for the opening the bony structures to gain access to the dural space (9, 18). Finally, it is of utmost importance to use the **micro Doppler probe** to insonate the major arteries (9, 18).

To increase the working space and the maneuverability of the instruments it is necessary: I) to remove the middle turbinate on one side; II) to lateralize the middle turbinate in the other nostril; and III) to remove the posterior portion of the nasal septum. A wider anterior sphenoidotomy, as compared to the standard approach, is performed, especially in lateral and superior directions where bony spurs are flattened in order to create an adequate space for the endoscope during the deeper steps of the procedure. All the septa inside the sphenoid sinus are removed including those attached on the bony protuberances and depressions on the posterior wall of the sphenoid sinus cavity. These surgical maneuvers allow the use of both nostrils, so that two or three instruments plus the endoscope can be inserted.

### Patient positioning

The patient is placed supine or in slight Trendelenburg’s position with the head turned 10°–15° on the horizontal plane, towards the surgeon, fixed in a rigid three-pin Mayfield-Kees skeletal fixation device in order to allow the use of the neuronavigation systems. On the sagittal plane, the head is extended for about 10–15 degrees to achieve a more anterior trajectory avoiding that either the endoscope and the surgical instruments hit on the thorax of the patient. The surgeon is on the patient's right side, in front of him. The endoscopic equipment and the neuronavigation system are positioned behind the head of the patient and in front of the surgeon (26–28).

## The transtuberculum transplanum approach to the suprasellar area

After the preliminary steps for extended transsphenoidal approaches have been performed, the bone removal over the sella starts with the drilling of the tuberculum sellae, which, from an inferior view, corresponds to the angle formed by the planum sphenoidale with the sellar floor. The drilling is then extended bilaterally, towards both the medial opto-carotid recesses. The extension of the bone removal depends on the size of the lesion and is performed under the control of neuronavigator. During the bone opening, it is not so rare to cause bleeding of the superior intercavernous sinus. In such cases it is preferable to close the sinus with the bipolar forceps instead of using the hemoclips, which narrows the dural opening: two horizontal incisions are made just few millimetres above and below the superior intercavernous sinus.

The dissection and the removal of the lesion in the suprasellar area follows the same principles of microsurgery and uses low-profile instruments and dedicated bipolar forceps. Also the strategy for tumor removal is tailored to each lesion, so that it will be different for giant pituitary adenomas, craniopharyngiomas or meningiomas.

## Approach to the ethmoidal planum

The endoscopic endonasal technique for the management of lesions of spheno-ethmoidal region has become popular for CSF leaks and, subsequently, for meningoencephaloceles and selected benign tumors of the anterior skull base (29–31). More recently the collaboration among ENT surgeons and neurosurgeons has brought advances in the use of the endoscopic endonasal technique in neurosurgery, thus permitting the pure endonasal treatment of intradural lesions, such as olfactory groove meningiomas or esthesioneuroblastomas (16).

Usually the bone of anterior skull base enclosed between the two orbits is removed, thus creating a surgical corridor, which can be extended laterally between the two medial orbital walls and antero-posteriorly from the frontal sinus to the sella, according to lesion extension. Once again, the intradural maneuvers are prerfomed according to conventional microsurgical principles.

## Approaches to the Cavernous Sinus and Lateral Recess of the Sphenoid Sinus

Different endoscopic endonasal surgical corridors have been described to gain access to different areas of the cavernous sinus (14, 32). These corridors have been related to the position of the intracavernous carotid artery (ICA). The first approach permits access to a compartment of the cavernous sinus medial to the ICA, while a second approach allows access to a compartment lateral to the ICA.

### Approach to the medial compartment of the cavernous sinus

It is indicated for lesions arising from the sella and projecting through the medial wall of the cavernous sinus, without extension into the lateral compartment. Actually it is mainly indicated in cases of pituitary adenomas. The procedure begins with the introduction of the endoscope through the nostril contralateral to the parasellar extension of the lesion. This because the endoscopic approach is paramedian and the view provided by the endoscope is much wider on the contralateral side. After the removal of the intra-suprasellar portion of pituitary adenoma, the intracavernous portion of the lesion is faced. The tumor itself enlarges the C-shaped parasellar carotid artery, thus making easier the suctioning and the curettage through this corridor. The completeness of the lesion removal is confirmed by the venous bleeding, easily controlled with irrigation and temporary sellar packing with haemostatic agents and/or cottonoids.

### Approach to the lateral compartment of the cavernous sinus and to the lateral recess of the sphenoid sinus

Is indicated in the case of tumors involving the entire cavernous sinus and arising from the sella, such as pituitary adenomas, or lesions coming from surrounding areas (middle cranial fossa, clivus, pterygopalatine fossa), such as chordomas and chondrosarcomas. This approach is ipsilateral to the parasellar extension of the lesion. The anterior wall of the sphenoid sinus and its septa are then widely removed in order to expose all the landmarks on the posterior wall of the sphenoid sinus. The bulla ethmoidalis and the anterior and posterior ethmoid cells are removed on the same side of the parasellar extension of the lesion, to create a wide surgical corridor between the nasal septum and the lamina papiracea.

The spheno-palatine artery is then isolated with bipolar coagulation or the use of hemoclips, if necessary. At this point the medial pterygoid process is removed, with the microdrill providing a direct access to the lateral recess of the sphenoid sinus (LRSS) and thus to the lateral compartment of the cavernous sinus.

Once the anterior face of the lesion has been exposed, before opening the dura, the use of Neuronavigation and the micro-Doppler is mandatory in identifying the exact position of the ICA. The tumor removal proceeds from the extracavernous to the intracavernous portion. The dura is then opened as far as possible from the ICA, which, in case of pituitary adenomas, allows the lesion to emerge under pressure. Delicate manouveres of curettage and suction usually allow the removal of the parasellar portion of the lesion, in the same fashion as for the intrasellar portion. Only after the removal has been completed will some bleeding begin, which is usually easily controlled with the use of hemostatic agents.

## Approach to the Clivus, Cranio-Vertebral Junction and anterior portion of the foramen magnum

The endoscope through the nose has been used for the management of clival lesions and more recently also for lesions located at the CVJ, either extradural (33–35) or intradural (34). The endoscopic endonasal approach provides the same advantages of direct route and minimal neurovascular manipulation offered by the transoral approach, but with a wider and closer view. Furthermore it avoids some of the disadvantages related to the transoral approach, such as the need for mouth retractors and splitting of the soft palate.

The access to the clivus needs a lower trajectory in respect to that necessary for the sellar region. After the preliminary steps (middle turbinectomy, removal of the posterior part of the nasal septum, wide sphenoidotomy) the procedure goes on with the removal of the inferior wall of the sphenoid sinus up to identify the Vidian nerves, that represent the lateral limits of the surgical corridor. The vomer and the inferior wall of the sphenoid sinus are completely removed, preserving the mucosa covering these structures, in order to create an useful mucosal flap for the closure of the surgical field. The bone of the clivus, according with the surgical necessity, is drilled and removed. At the level of the sphenoidal portion of the clivus the approach is limited laterally by the bony protuberances of intracavernous carotid artery. Furthermore it is important to highlight that the abducens nerve enters the cavernous sinus by passing through the basilar sinus medially than the paraclival tract of the intracavernous carotid artery. In case of lower extension of the lesion, it is possible to extend downward the bone removal up to the C2 vertebral body.

## Reconstruction techniques

Due to the consistent intraoperative CSF leakage resulting from a wider dural opening, an accurate reconstruction of the skull base defect results mandatory after lesion removal. The reconstruction should be watertight to prevent postoperative CSF leak, which could occur especially if lesion removal has required a large opening of the arachnoid cisterns or of the third ventricle.

Conventional sellar floor reconstruction techniques results inadequate during such extended approach for several reasons: i) the size of the defect; ii) the irregular shape of the defect due to the close distance of the osteo-dural defect to the optic nerves and carotids artery and finally iii) the wide intradural empty space lacking of arachnoidal barriers.

Thus far, the repair should proceed as for Grade 3 of Kelly’s paradigm (36) in order to achieve:

1. intradural sealing of the arachnoid;
2. watertight closure of the osteo-dural skull base defect;
3. packing of the sphenoid.

Recently, a variety of techniques for the reconstruction of skull base defects have been reported in the main literature (37); nevertheless, we found that reconstruction should mostly rely on the use of a vascularized flap, which provides faster healing and an earlier, more resilient seal .

In our experience, we have adopted three different reconstruction techniques, according with the different surgical conditions, namely the **Intradural reconstruction (so-called inlay),** the **Intra-extradural reconstruction (inlay-overlay)** and the **Extradural reconstruction (overlay).** According to this latter method, which figured out to be the most resilient and effective, a thin layer of fibrin glue (Tisseel^®^, Baxter, Vienna, Austria) is positioned in the intradural space as first barrier to CSF and more over to fill the dead space. The closure of the osteo-dural defect is then achieved, using a combination of a solid or semisolid buttress made of an easy to shape material such as a synthetic copolymer, i.e. LactoSorb (Walter Lorenz Surgical, Inc., Jacksonville, FL), with a dural substitute, i.e. Tutoplast^®^ (Tutogen Medical GmbH). A single layer of the dural substitute is positioned in the extradural space, covering the dural opening and the conformed sheath of the resorbable semi-solid material is then overlapped and embedded in the extradural space dragging the dural substitute in overlay position (37) .

Once a watertight barrier has been achieved multiple layers of dural substitute are placed over to support the reconstruction and/or a mucosal flap – usually a free muchoperichondrium flap harvested from the middle turbinate or from the nasal septum or a vascularized Hadad pedicled flap - is used to cover the posterior wall of the sphenoid sinus. Periombelical fat and fibrin glue are used to fill the sphenoid cavity, thus reducing “dead spaces” and to hold the material in place.

No lumbar drainage is employed at the end of the procedure.

## Conclusions

The endoscope has opened the eyes of the surgeon upon structures like the planum sphenoidale, the clivus, the carotid and optic bony protuberances, from upside down the common surgical view. Though, pathologies arising or extending in these regions – once approachable only with the more invasive transcranial surgery - such as craniopharyngiomas, tuberculum sellae meningiomas, macroadenomas involving the cavernous sinus, upper clival chordomas are candidate to be removed via the endonasal route. The endoscopic endonasal approach is gaining land everyday, thank to the interest of patients, cooperating surgeons from different specialities and technologic investors. Through the last two decades, an interesting interchange between the evolving approaches and the new technological advances has taken place.

Although at a first glance might be considered something that everyone can do, require an advanced and specialized training, both in the lab, with *ad hoc* anatomical dissections, and in the operating room; one should become familiar with the endoscopic skills, endoscopic anatomy and complication avoidance and management.

## Figures and Tables

**Figure 1:**
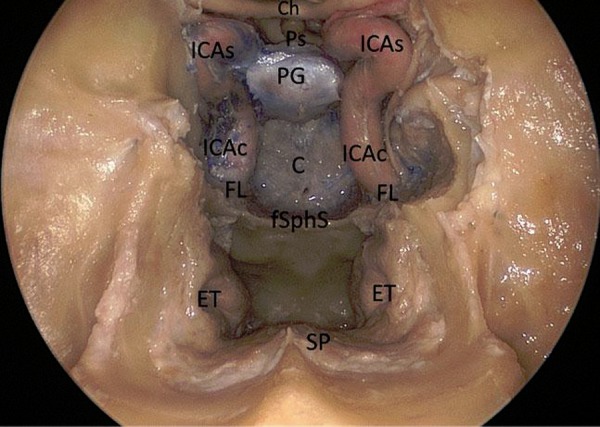
**Endoscopic endonasal anatomic picture showing the suprasellar, the parasellar and the clival area.** ***Ch: chiasm; Ps: pituitary stalk; PG: pituitary gland; C: clivus; fSphS: floor of the sphenoidal sinus; ET: Eustachian tube; SP: soft palate; FL: foramen lacerum; ICAc: paraclival segment of the internal carotid; ICAs: parasellar segment of the internal carotid artery.***

**Figure 2:**
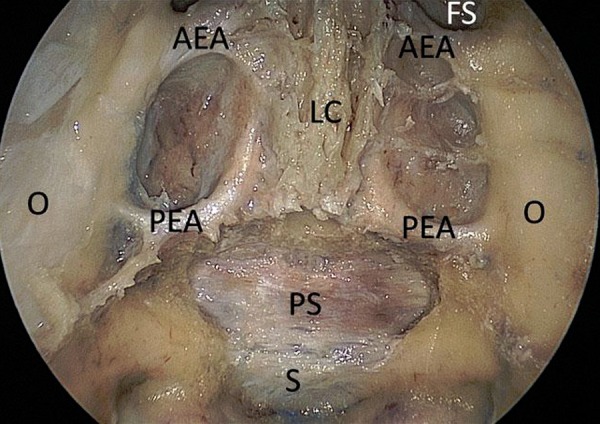
**Endoscopic endonasal anatomic view of the anterior skull base. The superior part of the nasal septum has been removed.** ***AEA: anterior ethmoidal artery; PEA: posterior ethmoidal artery; O: Orbit; LC: lamina cribriformis; PS: planum sphenoidale; FS: frontal sinus; S: sella turcica.***

**Figure 3:**
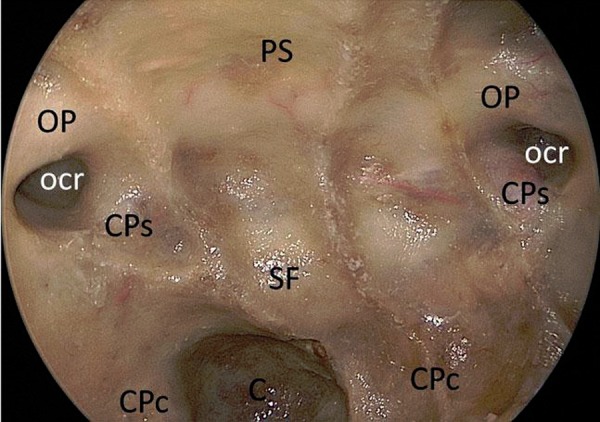
**Endoscopic endonasal anatomic view of the planum sphenoidale, the sellar floor and clival area.** ***PS: pituitary stalk; OP: optic protuberance; ocr: lateral opto-carotid recess; CPs: parasellar segment of the carotid protuberance; CPc: paraclival segment of the carotid protuberance.***

**Figure 4:**
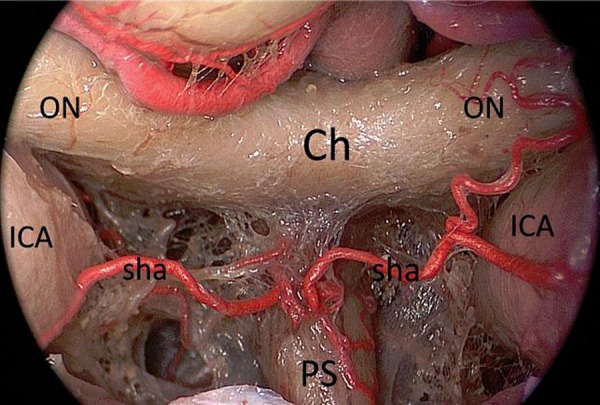
**Endoscopic endonasal anatomic close-up view of the chiasm, highlighting the superior hypophyseal arteries complex.** ***ICA: internal carotid artery; ON: optic nerve; Ch: chiasm; Ps: pituitary stalk; sha: superior hipophyseal artery.***

**Figure 5:**
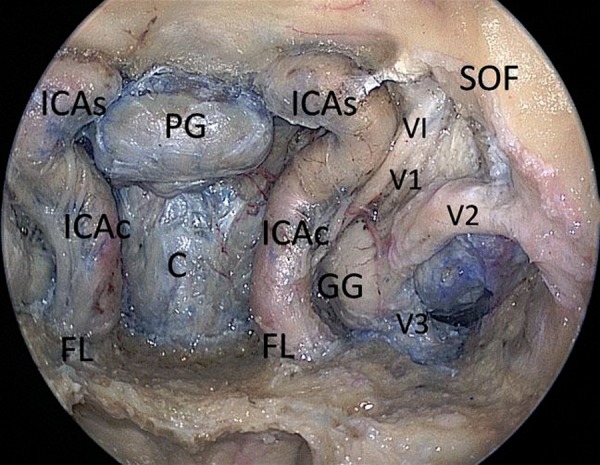
**Endoscopic endonasal cadaveric image: After the opening of the bony surfaces over the sellar, clival area and carotid protuberances the neurovascular structures of the left cavernous sinus have been exposed.** ***ICAc: paraclival segment of the internal carotid artery; ICAs: parasellar segment of the internal carotid artery; FL foramen lacerum; PG: pituitary gland; C: clivus; GG: Gasser’s ganglion; VI: abducent nerve; V1: first branch of trigeminal nerve; V2: second branch of trigeminal nerve; V3: third branch of trigeminal nerve; SOF: superior orbital fissure.***

**Figure 6:**
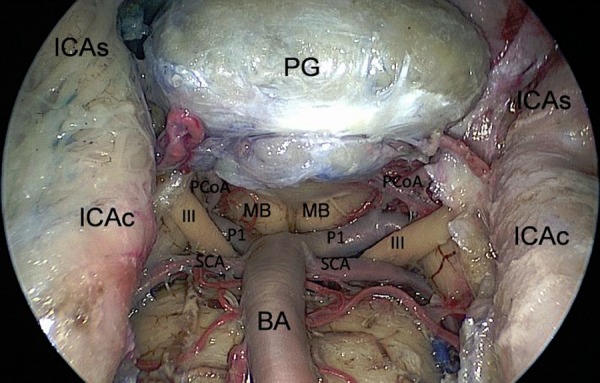
**Endoscopic endonasal view of the superior third of the retroclival area; exposure of the neurovascular structures, after bone removal.** ***ICAs: parasellar segment of the internal carotid artery; ICAc: paraclival segment of the internal carotid artery; III: third oculomotor nerve; SCA: superior serebellar artery; PCoA: posterior communicating artery; P1: pre-comunicating tract of the posterior cerebral artery; MB: mammilary body; PG: pituitary gland; BA: basilar artery.***

**Figure 7:**
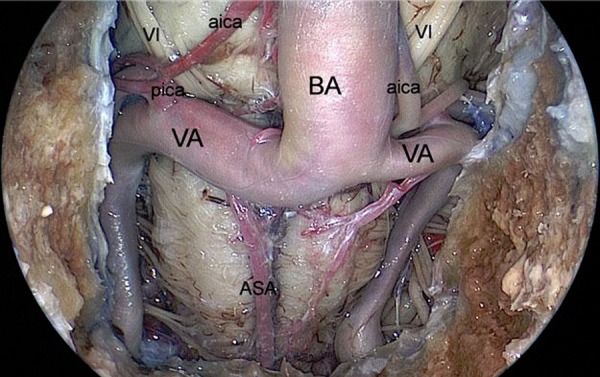
**Endoscopic endonasal view of the inferior third of the retroclival area.** ***BA: basilar artery; VA: vertebral artery; ASA: anterior spinal artery; pica: posterior inferior cerebellar artery; aica: anterior inferior cerebellar artery; VI: abducent nerve.***

**Figure 8:**
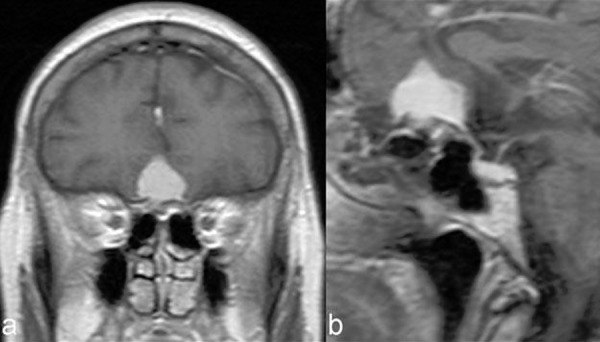
**Picture showing a preoperative MRI scan showing the case of a planum sphenoidale meningioma.**

**Figure 9:**
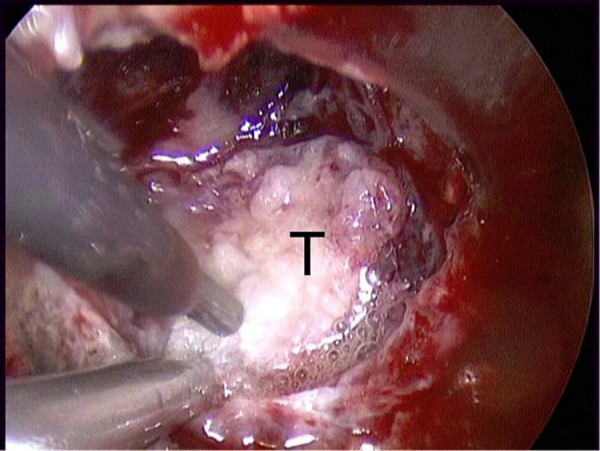
**Intraoperative image of piecemeal tumor removal using cavitational ultrasonic surgical aspiration (CUSA) of the planum sphenoidale meningioma shown in** figure 8. ***T: tumor.***

**Figure 10:**
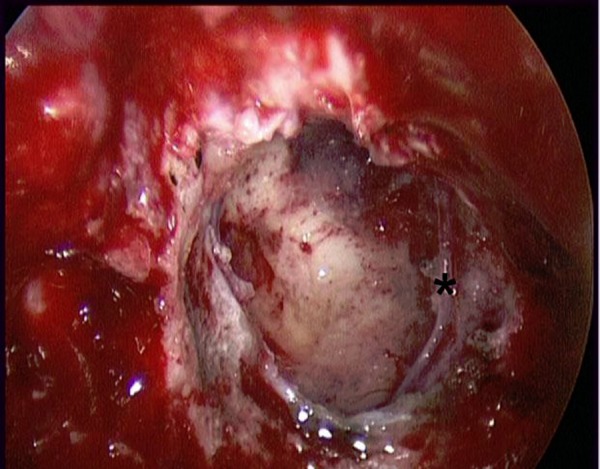
**Intraoperative image after tumor removal of the case shown in** figure 8. * branch of frontopolar artery.

**Figure 11:**
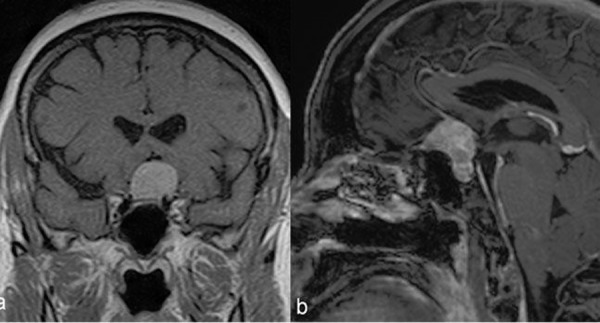
**Picture showing a preoperative MRI scan showing the case of a tuberculum sellae meningioma.**

**Figure 12:**
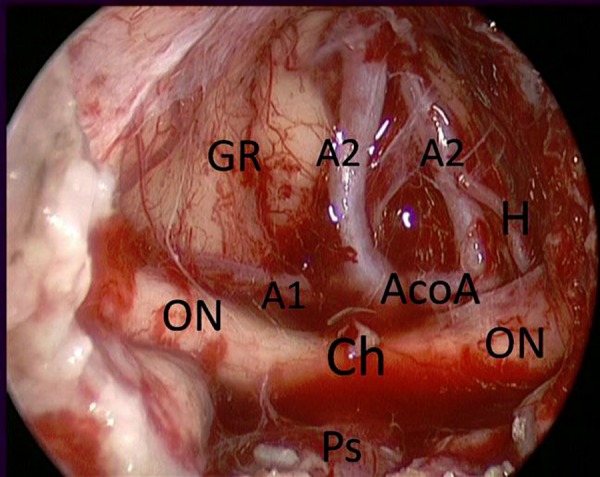
**Endoscopic endonasal intraoperative picture showing the suprachiasmatic area after tuberculum sellae meningioma (case in** figure 11) removal. ***GR: gyri recta; ON: optic nerve; A1: pre-communicating tract of anterior cerebral artery; A2: post-communicating tract of anterior cerebral artery; Ch: chiasm; Ps: pituitary stalk; AcoA: Anterior communicating artery; H: heubner artery.***
